# Integrating telemedicine and community support to enhance healthcare access for people with disabilities: a SANRA-aligned narrative review

**DOI:** 10.3389/fpubh.2026.1853385

**Published:** 2026-09-10

**Authors:** Osama Albasheer, Siddig Ibrahim Abdelwahab, Mayada Nogdalla, Afaf Hakami, Doaa Abdulwahab Mohammed Ayish, Fatma Ayish, Ali Yahya Maashi, Afnan Madkhali, Hatim Alessa, Ahmad Alharbi, Sameer Alqassimi, Mohammed Somaili, Mohammed Magry, Tahani Madkhali, Amal H. Mohamed

**Affiliations:** 1 Department of Family and Community Medicine, College of Medicine, Jazan University, Jazan, Saudi Arabia; 2 King Salman Center for Disability Research, Riyadh, Saudi Arabia; 3 Health Research Centre, Jazan University, Jazan, Saudi Arabia; 4 Family Medicine, Primary Healthcare, Ministry of Health, Riyadh, Saudi Arabia; 5 Department of Internal Medicine, Jazan General Hospital, Jazan, Saudi Arabia; 6 Department of Internal Medicine, College of Medicine, Jazan University, Jazan, Saudi Arabia; 7 Department of Family Medicine, Jazan University Hospital, Jazan University, Jazan, Saudi Arabia; 8 Department of Internal Medicine, Jazan Military Hospital, Jazan, Saudi Arabia; 9 Jazan Ministry of Health, Jazan, Saudi Arabia; 10 Department of Internal Medicine, Faculty of Medicine, Jazan University, Jazan, Saudi Arabia

**Keywords:** barriers, digital health, disabilities, healthcare, telemedicine

## Abstract

People with disabilities face multiple barriers to healthcare access globally, especially in low-resource and underserved settings. Telemedicine and community-based care have emerged as potential strategies to reduce these gaps, but evidence remains fragmented. This narrative review aimed to examine barriers to healthcare access for people with disabilities and explore the role of telemedicine and community-based interventions. A narrative review was conducted following SANRA guidelines. Searches were performed in PubMed, MEDLINE, Scopus, Web of Science, CINAHL, PsycINFO, and Google Scholar. Fifty studies were included from 116 records using *thematic* synthesis. Reporting followed SANRA to ensure transparency and thematic synthesis rather than quantitative pooling. Findings show that barriers are structural, attitudinal, financial, informational, and systemic. Rural populations face additional geographic and transport challenges. Telemedicine improves access by reducing distance and cost, while community-based strategies strengthen outreach and continuity of care. However, the digital divide and system limitations reduce effectiveness. Evidence also shows that inequities are more severe in LMICs and rural regions due to weak infrastructure and limited resources. Caregiver dependence and policy implementation gaps further limit autonomy and access despite existing legal frameworks. The review highlights the need for integrated, context-specific approaches combining policy reform, digital health, and community support to improve equitable healthcare access for people with disabilities. Future efforts should prioritize inclusive service design, digital equity, and strengthening health systems in underserved settings to improve health outcomes for all globally.

## 1 Introduction

Disability represents a major global public health challenge affecting approximately 1.3 billion people worldwide, according to the World Health Organization (WHO) Global Report on Health Equity for Persons with Disabilities (1, 2). The increasing prevalence of disability is associated with population aging, the growing burden of non-communicable diseases, injuries, and improved survival following acute health conditions. Despite advances in healthcare systems and disability rights frameworks, people with disabilities continue to experience substantial health inequities, including reduced access to preventive services, delayed diagnosis, inadequate treatment, and poorer health outcomes compared with people without disabilities (1, 3). These disparities are driven not only by individual health conditions but also by persistent structural, environmental, socioeconomic, and health-system barriers that influence healthcare access.

Barriers to healthcare access for people with disabilities are multifactorial and operate across structural, social, and systemic levels. Individuals with physical, sensory, intellectual, and psychosocial disabilities face difficulties in accessing both routine and specialized healthcare services (1, 4, 5). Physical barriers, such as inaccessible infrastructure and medical equipment, limit entry into healthcare facilities. At the same time, communication challenges, including the absence of accessible information formats and trained interpreters, restrict effective interaction with healthcare providers. These challenges are further compounded by limited provider knowledge and inadequate training in disability-inclusive care (6).

Beyond structural constraints, attitudinal barriers remain a critical concern. Stigma, discrimination, and misconceptions about disability often influence healthcare delivery and utilization. Negative assumptions about quality of life or functional capacity may lead to suboptimal care or exclusion from decision-making processes (7). For individuals with mental health or intellectual disabilities, these challenges are often intensified due to societal stigma and insufficient integration of mental health services within primary care systems (3). As a result, many individuals delay or avoid seeking care, leading to poorer health outcomes and widening health disparities.

These challenges are particularly pronounced in low- and middle-income countries (LMICs) and other underserved settings, where healthcare systems may face limitations related to workforce availability, infrastructure, financing, and service accessibility. Within this broader global context, Arab countries represent an important but relatively underexplored setting. The region includes diverse healthcare systems ranging from highly resourced health systems in Gulf countries to resource-constrained systems affected by economic instability and limited healthcare infrastructure. This variation provides an opportunity to examine how disability-related healthcare barriers and digital health solutions operate across different levels of health system capacity. Although several studies have explored disability inclusion and healthcare access within specific Arab countries, evidence remains fragmented and often focuses on individual barriers rather than examining the interaction between structural inequities, emerging digital health approaches, and community-based strategies. In addition, the rapid expansion of telemedicine following the COVID-19 pandemic has created new opportunities for improving healthcare access but has also introduced challenges related to digital accessibility, infrastructure, and equity. Therefore, an integrated synthesis examining both barriers and potential solutions across diverse contexts is needed. While there have been notable improvements in healthcare infrastructure and service delivery across the region, equitable access for people with disabilities remains limited (8, 9). Structural barriers such as inadequate transportation systems, uneven distribution of healthcare facilities, and high out-of-pocket costs continue to restrict access, particularly in rural and remote areas (10).

Cultural perceptions of disability further shape access to care in the region. In some contexts, disability is associated with stigma or social exclusion, which may discourage individuals from seeking healthcare services or participating fully in society (11, 12). In addition, socioeconomic factors, including higher rates of unemployment, poverty, and limited educational opportunities among people with disabilities, exacerbate existing inequities and contribute to poorer health outcomes (12, 13). These intersecting challenges highlight the need for context-specific and inclusive healthcare approaches that address both systemic and sociocultural determinants of access.

In response to these challenges, there has been increasing interest in alternative and complementary models of healthcare delivery. Telemedicine has emerged as a promising strategy to improve access by reducing geographical and physical barriers, enabling remote consultations, and facilitating continuity of care (14, 15). However, digital health interventions operate within existing social and structural inequalities. Access to telemedicine is not determined solely by the availability of technology but also by individuals’ ability to obtain devices, maintain reliable connectivity, develop digital skills, and use platforms that accommodate different functional abilities. Therefore, the digital divide extends beyond a simple gap in technology ownership and represents a broader form of digital exclusion shaped by disability, socioeconomic status, education, geography, and healthcare infrastructure. For people with disabilities, this distinction is particularly important because a platform may be technically available but remain inaccessible or difficult to use due to inadequate accessibility features, such as lack of screen-reader compatibility, captioning, alternative communication formats, or adaptable interfaces. Similarly, community-based approaches, including community health workers and local support systems, have shown potential in addressing these barriers and improving engagement with healthcare services (16–18). These approaches are particularly relevant in underserved settings, where traditional healthcare delivery models may be insufficient or inaccessible.

Despite the growing body of literature on disability and healthcare access, existing evidence remains fragmented across disciplines, geographical regions, and intervention approaches. Previous reviews have often examined disability-related healthcare barriers, digital health implementation, or community-based interventions independently, limiting understanding of how these factors interact within different healthcare contexts. Furthermore, evidence from Arab countries remains comparatively limited within global disability and digital health discussions, despite substantial variation in healthcare resources, sociocultural environments, and policy frameworks across the region. There is a need for an integrative synthesis that brings together insights on barriers to care alongside emerging strategies such as telemedicine and community-based interventions, with attention to regional and contextual variations. This narrative review addresses this gap by integrating evidence on healthcare access barriers among people with disabilities with emerging strategies, including telemedicine and community-based approaches. By incorporating evidence from both high-income and LMIC settings, with particular attention to Arab and underserved contexts, this review aims to provide a broader understanding of how healthcare systems can develop more inclusive and context-responsive models of care. This narrative review aimed to examine the key barriers to healthcare access for people with disabilities and to explore the role of telemedicine and community-based strategies in addressing these barriers, with particular consideration of underserved settings and the Arab region.

## 2 Methods

This narrative review was conducted in accordance with the principles of the Scale for the Assessment of Narrative Review Articles (SANRA), emphasizing methodological transparency, justification of the literature search strategy, and critical synthesis of available evidence. The review was designed as a narrative synthesis of diverse evidence sources rather than a systematic review, scoping review, or meta-analysis. Therefore, the objective was not to identify all available evidence, quantitatively synthesize findings, or estimate intervention effects, but rather to critically interpret and integrate evidence from disability studies, digital health research, healthcare access literature, and community-based intervention frameworks.

A structured literature identification process was applied to improve transparency and traceability of the included evidence. However, this process should not be interpreted as a PRISMA-based systematic screening procedure. The numerical description of identified and included records was used only to demonstrate the scope of literature considered and to provide transparency regarding evidence selection within a narrative review framework.

### 2.1 Search strategy

A structured but flexible literature search was undertaken to identify relevant studies addressing healthcare access for people with disabilities, with particular focus on telemedicine and community-based strategies. Electronic databases, including PubMed, MEDLINE, Scopus, Web of Science, CINAHL, PsycINFO, and Google Scholar, were searched. The search was designed to capture both empirical and conceptual literature. Emphasis was placed on studies published within the last 10 years (2015–2025) because this period corresponds with substantial global expansion in digital health infrastructure, increased adoption of telemedicine platforms, and growing policy attention toward technology-enabled healthcare delivery. Furthermore, the COVID-19 pandemic accelerated the implementation of telemedicine and highlighted both its potential and limitations in maintaining healthcare access for underserved populations, including people with disabilities. However, this timeframe was not applied as an absolute exclusion criterion. Earlier foundational studies were considered when they provided essential theoretical frameworks, established evidence regarding disability-related healthcare barriers, or contributed important policy perspectives relevant to current healthcare access challenges. Therefore, the review prioritized contemporary evidence while retaining selected earlier literature where necessary to provide historical and conceptual context.

The use of a recent publication window may introduce potential bias by increasing representation of pandemic-era digital health research, particularly studies examining rapid telemedicine adoption during and after COVID-19. To address this limitation, findings were interpreted considering broader healthcare access literature, including pre-pandemic evidence on structural, social, financial, and systemic barriers affecting people with disabilities.

To improve coverage, backward and forward citation tracking was performed on key articles, and reference lists of relevant reviews and policy reports were manually screened to identify additional pertinent studies not retrieved through database searches. Although this narrative review incorporated multiple international databases, citation tracking, and diverse evidence sources to enhance the breadth of the literature captured, the selection of available evidence may still be influenced by publication and database availability biases. Evidence from LMICs and the Arab region may be underrepresented because locally generated research, policy documents, and non-indexed publications are less consistently available in international databases. Relevant reports and policy documents were considered when they contributed important contextual insights into disability inclusion, digital health implementation, and healthcare accessibility; however, variations in publication practices and accessibility of regional literature may have affected the visibility of certain perspectives. Additionally, the restriction to English-language literature may have limited the inclusion of relevant evidence published in other languages, particularly Arabic and French, which may be important for understanding disability and healthcare access within specific regional contexts.

### 2.2 Search keywords

The search strategy incorporated a combination of keywords and Boolean operators to capture relevant literature. Core search terms included: “disability,” “people with disabilities,” “healthcare access,” “healthcare barriers,” “telemedicine,” “telehealth,” “digital health,” “community-based care,” “community health services,” and “health inequities.” These terms were combined using Boolean operators (AND/OR) and adapted across databases to ensure comprehensive retrieval of relevant studies. Variations and synonyms of key terms were also used to maximize the sensitivity of the search.

### 2.3 Study identification and selection process

A transparent literature identification process was undertaken to support methodological clarity within the narrative review framework. A total of 116 records were identified through database searches and additional sources. These records were reviewed for relevance to the review objectives, focusing on healthcare access among people with disabilities and the role of telemedicine and community-based strategies. Rather than applying systematic review eligibility procedures, the selection process involved assessment of conceptual relevance, contribution to the review questions, and appropriateness of evidence for narrative synthesis.

**Figure 1:**
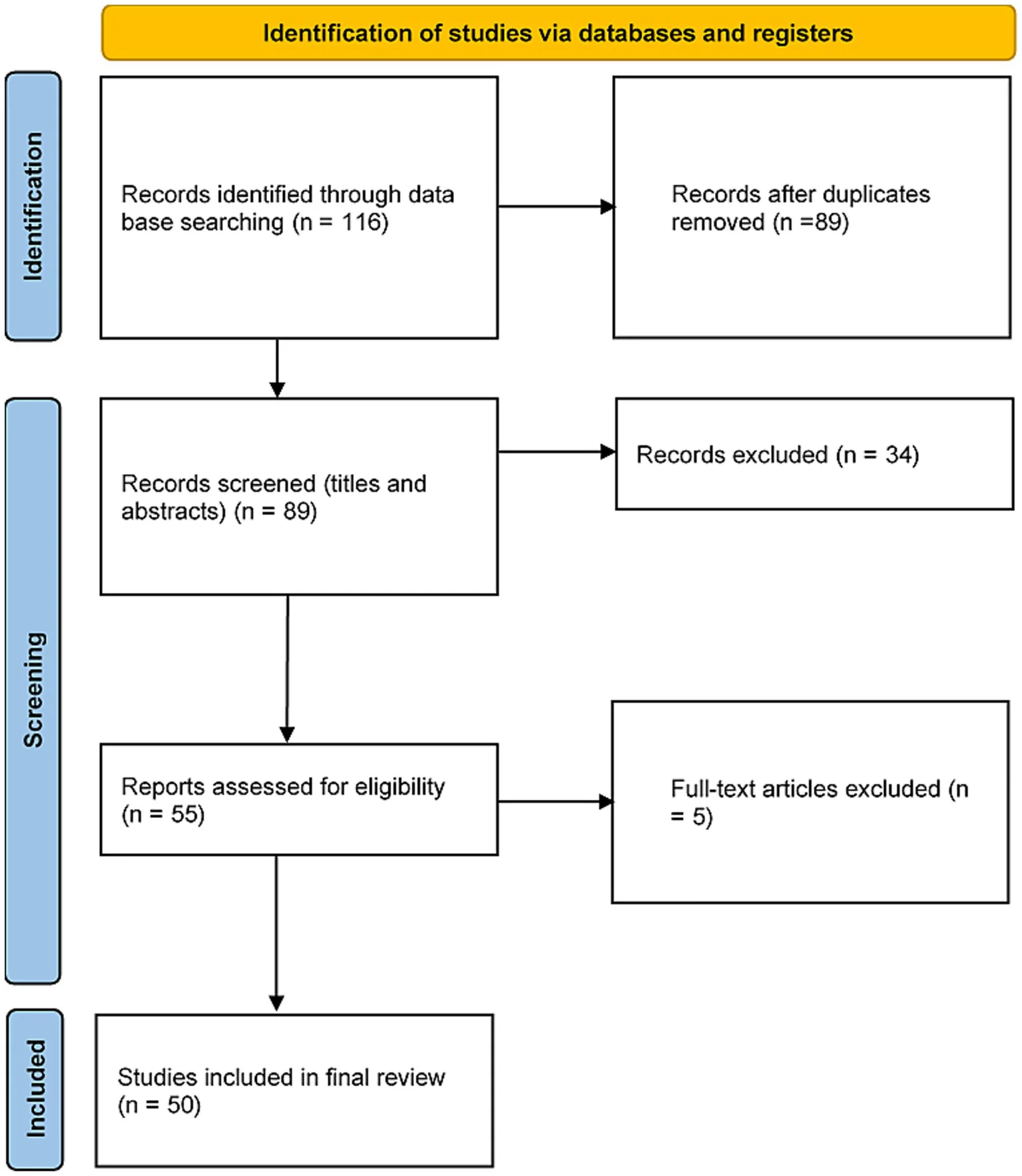
Literature identification and study selection process for the narrative review.

Records were excluded when they did not address disability-related healthcare access, healthcare barriers, telemedicine, digital health, or community-based approaches. Full-text assessment was conducted to determine whether studies provided meaningful insights relevant to the thematic objectives of the review. Following this process, 50 studies representing empirical research, systematic and scoping reviews, policy documents, and conceptual literature were included in the narrative synthesis. The included evidence comprised qualitative studies, observational research, systematic and scoping reviews, policy documents, and conceptual papers, allowing exploration of healthcare access challenges and intervention strategies across different healthcare settings. The selection process is presented in Figure 1 as a literature identification and screening overview. Given the narrative review design, studies were not subjected to formal risk-of-bias assessment or quantitative quality scoring. Instead, evidence was critically interpreted according to study design, methodological approach, contextual relevance, and contribution to the review objectives.

### 2.4 Inclusion criteria

Studies were included if they:

- Examined healthcare access among people with disabilities
- Identified barriers to healthcare access (e.g., physical, attitudinal, financial, or systemic)
- Explored or evaluated telemedicine, telehealth, or community-based healthcare interventions
- Provided empirical findings, conceptual insights, or policy-level analysis relevant to the topic
- Were published in peer-reviewed journals or as credible gray literature (e.g., policy briefs, organizational reports)
- Were available in English. Due to resource limitations, studies published in languages other than English were not included.

### 2.5 Exclusion criteria

Studies were excluded if they:

- Focused on populations without disabilities
- Did not address healthcare access or related barriers
- Were not relevant to telemedicine or community-based approaches
- Were editorial pieces, commentaries, or opinion articles lacking substantive analysis
- Had insufficient methodological detail or limited relevance to the review objectives
- Were duplicates or unavailable in full text

### 2.6 Data extraction and synthesis

Data from the included studies were extracted and organized thematically. Key information included study context, population characteristics, type of disability addressed, methodological design, identified barriers to healthcare access, and reported interventions or strategies. The extracted data were synthesized using a narrative thematic approach, guided by the principles outlined by Wong et al. (19) and Popay et al. (20). Studies were grouped into thematic categories reflecting major domains of healthcare barriers (e.g., physical, attitudinal, logistical, financial, and systemic) and corresponding intervention strategies, including telemedicine and community-based care.

The synthesis approach prioritized interpretation of similarities, differences, and contextual influences across evidence sources rather than ranking studies according to methodological hierarchy or generating pooled estimates. This approach allowed for an integrative interpretation of evidence, with particular attention to variations across geographical regions, including underserved settings and the Arab world.

## 3 Results

### 3.1 Study selection and characteristics

The literature search yielded a total of 116 records across selected databases. After removal of duplicates and clearly irrelevant records, 89 studies remained for title and abstract screening. During this stage, 34 records were excluded due to a lack of relevance to disability-related healthcare access or the absence of focus on telemedicine and community-based strategies. Following this, 55 full-text articles were assessed for eligibility. Of these, 5 studies were excluded due to insufficient methodological clarity, lack of relevance to the research objective, or duplication of findings across publications. Ultimately, 50 studies were included in the narrative synthesis (Figure 1). The selection process represents a transparent overview of literature identification and screening rather than a PRISMA-based systematic review process. The included studies comprised a diverse body of literature, including observational studies, qualitative research, systematic and scoping reviews, policy reports, editorials, and conceptual papers, reflecting the interdisciplinary nature of disability-inclusive healthcare research. The studies represented both high-income and low- and middle-income countries (LMICs), with particular representation from underserved settings and the Arab region. In addition to database searching, manual screening of reference lists and key publications was conducted to identify relevant studies not captured in the initial search. A table of included studies is presented as Table 1.

**Table 1:** Summary of included studies.

Author	Year	Study type	Country	Population	Key focus	Key findings
Clemente et al. (1)	2022	Scoping review	Global	People with disabilities (PWDs), healthcare providers	Identification of barriers to healthcare access	Multiple barriers identified: communication gaps, financial constraints, attitudinal issues, transport and organizational barriers. Providers reported lack of training, system failures, and resource limitations. Highlights mismatch between user and provider perspectives.
McBride-Henry et al. (5)	2023	Scoping review	Global	PWDs, caregivers during COVID-19	Experiences of healthcare access during the pandemic	Reduced access led to feelings of invisibility and negative mental health outcomes. Policies were largely “disability-blind.” Individuals with multiple vulnerabilities faced greater exclusion. Calls for inclusive, disability-responsive systems.
Dassah et al. (6)	2018	Systematic Review (Framework Synthesis)	Global (mostly LMICs)	PWDs in rural areas	Determinants of access to primary healthcare (PHC)	Access shaped by availability, affordability, acceptability, and geography. Rural populations faced long travel distances, transport issues, and unaffordable services. Emphasizes need for integrated policy and provider training.
Hashemi et al. (11)	2022	Meta-synthesis (Qualitative Studies)	LMICs (multi-country)	PWDs	Barriers to primary healthcare access	Barriers categorized into attitudinal/cultural, informational, and logistical. Barriers interact dynamically, influencing care-seeking behavior and quality of care. Emphasizes need for holistic and system-level interventions.
Zuurmond et al. (61)	2019	Qualitative study	Cameroon and India	Adults with visual, hearing, and physical impairments	Multi-level factors influencing healthcare access	Access influenced by individual, household, community, and system-level factors. Key issues: stigma, economic constraints, poor referral systems, and trust deficits. Intersectionality (gender, age) shapes access. Recommends multi-layered interventions.
Eide et al. (21)	2015	Cross-sectional (Population-based survey)	Sudan, Namibia, Malawi, South Africa	General population incl. PWDs (*n* = 9,307)	Magnitude and predictors of access barriers	Major barriers: transport, service availability, lack of drugs/equipment, and cost. Barriers vary by socio-economic status, urbanity, and severity of disability. Highlights inequity—PWDs face compounded disadvantages beyond general population barriers.
Munthali et al. (24)	2019	Qualitative Study	Malawi	PWDs (multiple impairments)	Lived experiences of healthcare access barriers	Key barriers: cost, long distance, lack of transport, inaccessible terrain, poor provider attitudes, and communication gaps. Stigma and discrimination remain central. Recommends outreach services, assistive devices, and disability-friendly systems.
Tesfaye et al. (25)	2021	Cross-sectional Study	Ethiopia	Physically disabled individuals (*n* = 345)	Accessibility and associated factors	Only 25.4% had adequate access; majority faced physical, equipment, and communication barriers. Access improved with NGO support, absence of discrimination, and financial coverage. Emphasizes role of economic support and inclusive systems.
Coombs et al. (26)	2021	Cross-sectional (Population-based)	USA	Adults with mental health challenges (*n* = 50,103)	Prevalence and determinants of healthcare access barriers	95.6% reported ≥1 barrier, mainly affordability-related. Social determinants (income, ethnicity, employment benefits) strongly influenced access. Mental health burden intersects with systemic inequities rather than acting independently.
Ordway et al. (60)	2021	Mixed Methods Study	USA	PWDs, policymakers, stakeholders	Effectiveness of disability legislation (ADA) in healthcare access	Persistent barriers despite legal frameworks. ADA alone insufficient; partial improvements linked to ACA policies. Highlights gap between policy and implementation, emphasizing need for enforcement and systemic reform.
Rotarou & Sakellariou (12)	2017	Cross-sectional study (secondary data analysis)	Chile	7,459 adults with disabilities vs. 68,695 without	Inequalities in healthcare access despite universal health coverage	People with disabilities had significantly worse access (appointments, transport, cost, medicines); universal health coverage did not ensure equitable access; economic and systemic barriers persisted
Grills et al. (22)	2017	Cross-sectional population-based survey	India	2,431 adults (with and without disabilities)	Disability prevalence and barriers to service access	People with disabilities had significantly lower access; key barriers included lack of information, transport issues, and physical inaccessibility; socio-demographic factors influenced access
Marella et al. (23)	2018	Qualitative study	Indonesia	Stakeholders (*n* = 27) and people with disabilities (*n* = 12)	Factors influencing disability inclusion in eye health services	Barriers linked to infrastructure, provider attitudes, lack of training, cost, transport, and weak policy support; inclusion requires system-level planning and stakeholder engagement
Kabia et al. (35)	2018	Qualitative (intersectional analysis)	Kenya	Women with disabilities living in poverty	Impact of gender, disability, and poverty on access to healthcare	Intersectionality worsens access; transport costs, need for assistance, negative attitudes, and inaccessible facilities led to underutilization even when services were free
Soltani et al. (37)	2017	Qualitative study	Iran	People with disabilities, providers, policymakers (*n* = 50)	Cultural barriers to healthcare access	Cultural stigma, discrimination, provider reluctance, and lack of policy attention were major barriers; misconceptions about disability existed at all societal levels
Anawade et al. (30)	2024	Comprehensive review	Global	General population (various settings)	Impact of telemedicine on healthcare accessibility	Telemedicine improves access by reducing geographical, financial, and temporal barriers; enhances efficiency and equity; however, technological, regulatory, and user-acceptance barriers persist
Haleem et al. (31)	2021	Narrative review	Global	General population (patients and providers)	Capabilities and barriers of telemedicine	Telemedicine reduces travel cost and time, improves follow-up and access during COVID-19; barriers include technological limitations and need for infrastructure; complements but does not replace in-person care
Shigekawa et al. (34)	2018	Rapid review (systematic reviews and meta-analyses)	Global	Patients across multiple clinical areas	Effectiveness and impact of telehealth	Telehealth is generally equivalent to in-person care in many conditions; impact on healthcare utilization is unclear; effectiveness depends on context, population, and implementation factors
Yen Hwei & Octavius (32)	2021	Literature review	Global	Patients, healthcare providers, hospitals	Advantages and disadvantages of telemedicine	Benefits include remote access and efficiency; limitations include provider resistance, legal/regulatory uncertainty, and implementation challenges across systems
Shokri et al. (44)	2023	Narrative review	Global	General population (pandemic context)	Role of telemedicine in infectious disease pandemics	Telemedicine reduces infection spread, lowers healthcare burden, and improves access during pandemics; supports remote care and communication; cost-effective but dependent on technology availability
Portnoy et al. (33)	2020	Editorial	USA	General patients (COVID-19 context)	Role of telemedicine during COVID-19	Telemedicine supports triage, reduces infection risk, and maintains care access; barriers include low awareness, patient preference for familiar providers, and limited adoption despite policy support
Valdez et al. (39)	2021	Commentary / Policy analysis	USA (generalizable)	People with disabilities	Telehealth equity for people with disabilities	Telehealth can reduce barriers but may worsen inequities if not inclusively designed; lack of accessible technology and policy gaps risk excluding disabled populations.
Annaswamy et al. (58)	2020	Commentary / Review	Global	People with disabilities	Barriers to telemedicine for disabled populations	Persistent barriers include infrastructure, communication, regulatory, and accessibility issues; telemedicine adoption exposed new long-term challenges for disabled individuals.
Khoong et al. (38)	2020	Commentary/Perspective	USA	Vulnerable populations (low-income, older adult, minorities)	Equity in telemedicine for chronic disease management	Rapid telemedicine expansion risks increasing disparities due to the digital divide, low literacy, and language barriers; it requires policy and system-level interventions for equitable access
Edwards et al. (54)	2014	Cross-sectional survey	UK	Patients with chronic diseases (depression, CVD risk)	Patient interest in telehealth	Moderate interest in phone/email-based telehealth; acceptance driven by confidence in technology and perceived benefits; social media-based telehealth had low acceptance.
Serper & Volk (43)	2018	Systematic Review	USA (mainly)	Patients with chronic liver disease	Role of telemedicine in chronic disease management and access to specialist care	Telemedicine improves access to specialist care (e.g., rural, incarcerated populations) and shows similar or better clinical outcomes (e.g., HCV treatment). High patient satisfaction. However, major barriers include regulatory restrictions, reimbursement issues, and infrastructure gaps, limiting large-scale implementation.
Rutledge et al. (42)	2017	Literature Review	USA	Nurse practitioners and students	Telehealth training and workforce readiness	Telehealth improves access, especially in underserved areas, but a lack of provider training is a major barrier. Multimodal training (simulation, practice) improves provider competence. Highlights workforce readiness as a key determinant of telehealth success.
Pappas et al. (57)	2019	Qualitative Study	UK	Patients, GPs, nurses, consultants	Decision-making and interaction in telemedicine	Telemedicine enhances interprofessional collaboration and provider involvement, but may limit patient participation in decision-making. Raises concern about patient-centered care quality in digital consultations.
Henderson et al. (55)	2014	Brief report	USA	General healthcare population	Role of nurse practitioners in telehealth	Telehealth improves access, cost-effectiveness, and care coordination, especially for remote populations. Nurse practitioners play a key role in bridging access gaps. However, implies need for scalable and sustainable system models.
Sauers-Ford et al. (53)	2019	Qualitative study	USA	Pediatric clinicians, nurses, stakeholders	Adoption and usability of telemedicine in pediatric emergency care	Telemedicine improves collaboration and patient/family involvement, but adoption is limited due to provider bias, resistance, and workflow issues. Technology adaptability and user acceptance are critical for success.
World Health Organization (2)	2016	Report/Review	Eastern Mediterranean Region (multiple countries)	Community health workers (CHWs) and general population	Role of CHWs in improving access to primary health care	CHWs improve access, reduce mortality, enhance service coverage, and are cost-effective in low-resource settings
Shrestha et al. (18)	2024	Narrative Review	South Asia (multi-country)	Community Health Workers (CHWs) and populations	Role of CHWs in strengthening primary health care (PHC)	CHWs improve community-level access, health education, and maternal/child services. However, effectiveness depends on integration into health systems, training, and support. Weak system support limits scalability despite strong potential for UHC.
Khatri et al. (16)	2024	Scoping Review	Global (multi-country)	Community health programs (CHPs)	CHPs in achieving equity and UHC	CHPs improve access, affordability, and community-based care, and address social determinants of health. Key barriers include limited funding, poor service quality, weak private sector engagement, and neglect of NCDs, highlighting system-level gaps.
National Academies (51)	2017	Review/Report	USA	General population	Social determinants and community-based solutions for health equity	Health inequities are driven by structural and social determinants (poverty, housing, education, transport). Healthcare access alone is insufficient—multi-sectoral policy action is required to reduce disparities.
Cruz et al. (50)	2017	Literature Review	LMICs	Children (under 5)	Impact of conditional cash transfers (CCTs) on health equity	CCTs improve immunization and child morbidity outcomes, but effects on mortality are inconsistent. Financial support alone is insufficient if health system quality is poor, showing the limits of economic interventions alone.
Jamal et al. (45)	2022	Scoping Review	LMICs (global)	General population	Social Health Insurance (SHI) for UHC	SHI improves financial protection and access, but faces major barriers: implementation challenges, inequality in coverage, and system inefficiencies. UHC requires combined financing approaches (tax + insurance) rather than SHI alone.
Zhang et al. (47)	2024	Cross-sectional study	China	People with disabilities	Association between health insurance and rehabilitation service utilization	Health insurance (especially higher-benefit schemes) is associated with increased rehabilitation service use. Community-level service availability also improves utilization. Highlights financial coverage as a key enabler, but disparities exist between insurance schemes, indicating inequity within systems.
Mitra et al. (46)	2017	Systematic review	Multi-country (mostly high-income)	People with disabilities	Extra costs of living with disability	Disability is associated with high additional direct and indirect costs, especially for severe disability and smaller households. Evidence is limited in LMICs, indicating a major research gap. Financial burden contributes to poverty and inequity.
Thomas et al. (49)	2022	Case study + policy/ethical review	USA	Family caregivers	Financial burden in caregiving	Caregivers face economic, employment, and ethical challenges. Financial strain affects both caregiver well-being and patient care access. Emphasizes the need for policy support (e.g., leave, compensation) and the nursing role in mitigating burden.
Lamech et al. (48)	2019	Qualitative study	India	Family caregivers of dementia patients	Caregiver needs and health system gaps	Caregivers face physical, psychological, social, and financial strain. Key needs include trained health workers, affordable services, and better information. Highlights health system limitations and cultural influences on care-seeking behavior.
Barbareschi et al. (4)	2021a	Qualitative (focus groups + secondary analysis)	Kenya	People with disabilities & the general population	Disability stigma and discrimination	Stigma is driven by social, religious, and cultural beliefs. Assistive technology can both reduce barriers and unintentionally increase stigma by “marking” disability. Social attitudes remain a major barrier to inclusion and service uptake.
Sahu and Sahu (36)	2015	Editorial/Observational narrative	India	People with disabilities	Attitudinal barriers	Identifies attitudinal barriers as the most fundamental barrier influencing other types (physical, systemic). Describes forms such as pity, inferiority, stereotyping, hero worship, denial, and ignorance. These attitudes lead to social exclusion and discrimination, even when physical access exists. Emphasizes that equal opportunities depend heavily on social attitudes and inclusion rather than legislation alone. Highlights how supportive environments can reduce disability impact, while negative attitudes worsen functional limitations.
Oluyede et al. (27)	2022	Qualitative study (interviews)	USA (North Carolina)	Care coordinators (health professionals)	Transportation barriers & telehealth	COVID-19 exacerbated existing transportation barriers and created new ones. Telehealth emerged as a key mitigation strategy, improving access to mental health services, continuity of care, and reduced exposure risk. However, telehealth is not suitable for all services (e.g., emergency care). Highlights how system-level innovations (telehealth) can partially offset structural barriers like transportation.
Al Shamsi et al. (52)	2020	Systematic review	Multi-country	Patients and healthcare providers	Language barriers in healthcare	Language barriers lead to miscommunication, reduced patient satisfaction, lower quality of care, and compromised patient safety. Interpreter services improve communication but increase cost and duration of care. Online translation tools (e.g., Google Translate, MediBabble) improved satisfaction (~92%) and care quality. Demonstrates how communication barriers directly affect healthcare outcomes and system efficiency.
Panezai et al. (7)	2017	Cross-sectional survey	Pakistan	General population (302 respondents)	Gender-based access to PHC	Significant gender disparities and systemic barriers in accessing primary healthcare. Barriers include distance, transportation, income, staff availability, service hours, and facility location. Women accessed services more frequently but overall utilization was low. Highlights need for gender-sensitive health policies and improved service organization to enhance equitable access.
Kronfol (28)	2012	Narrative review	Arab countries (regional)	General population	Health system barriers	Identifies barriers as social, cultural, financial, administrative, and organizational, affecting different groups unequally (especially gender and ethnic groups). Calls for policy interventions to reduce inequities and improve access through equity-oriented health systems and social policies. Emphasizes structural and governance-level determinants of access.
Katoue et al. (9)	2022	Narrative/scoping review	MENA region	Health systems (policy level)	Health system development and barriers	MENA health systems face inequitable access, workforce shortages, rising chronic disease burden, weak health information systems, and leadership challenges. Reforms focus on financing, regulation, organization, and provider behavior. Highlights that structural and governance issues remain major barriers to equitable access, despite ongoing reforms. Emphasizes need for stakeholder collaboration and system-wide improvements.
Mani and Goniewicz (29)	2024	Rapid review	Saudi Arabia	National healthcare system	Healthcare transformation (Vision 2030)	Vision 2030 has driven major reforms in infrastructure, digital health, workforce development, and quality improvement. Focus on improving accessibility, efficiency, and patient safety. Demonstrates a shift toward innovation-driven, equity-oriented healthcare systems, positioning digitalization as a key enabler of access.
AlWatban et al. (56)	2024	Book chapter/review	Saudi Arabia	Digital health ecosystem	Digital health transformation	Rapid expansion of digital health technologies, including EHRs, telemedicine, mHealth, AI, virtual reality, and virtual hospitals. Key stakeholders include MOH, providers, and patients. Digital health improves service accessibility, continuity of care, and system efficiency, particularly in low-resource or remote settings. Highlights technology as a solution to structural and geographic barriers, but implies dependence on infrastructure and digital literacy.
Wilson et al. (40)	2021	Qualitative study (interviews)	Canada (rural setting)	Rural family physicians	Communication barriers in referrals	Identifies communication challenges between rural physicians and urban specialists. Key barriers include geographical isolation, referral delays, lack of contextual understanding, and communication gaps. Emphasizes the importance of respectful, context-aware communication for effective care coordination. Highlights that poor communication can function as an indirect barrier to timely and appropriate healthcare access.

### 3.2 Thematic synthesis of findings

Across the included studies, healthcare access barriers were not identified as isolated challenges but as interconnected mechanisms operating across individual, community, and health-system levels. A consistent pattern emerged whereby improvements in one domain were frequently limited by constraints in another. For example, digital health interventions reduced geographical barriers but did not consistently improve access among individuals experiencing digital poverty, inaccessible technology, or limited support systems. Similarly, community-based approaches improved engagement and trust but demonstrated variable scalability depending on workforce availability and long-term funding. These findings suggest that healthcare accessibility is shaped less by the presence of individual interventions and more by the interaction between intervention design and surrounding social, economic, and health-system conditions. Across the included evidence, the most frequently recurring barriers were structural and logistical barriers, including inaccessible facilities, transport difficulties, distance, and weak service distribution, followed by financial constraints, attitudinal barriers, informational and communication barriers, and broader policy implementation gaps. Telemedicine and community-based care were therefore interpreted not as isolated solutions but as strategies whose relevance depends on the dominant barriers within each setting.

#### 3.2.1 Physical and structural barriers

Physical barriers were consistently reported across the included studies as a major determinant of limited healthcare access. These include inaccessible infrastructure, lack of assistive facilities, and poorly adapted healthcare environments, as highlighted in multiple global and LMIC-based studies (12, 21–25). Rural and underserved populations were particularly affected due to long distances, inadequate transportation, and limited facility distribution (6, 26, 27). Structural inequities within healthcare systems further exacerbate these challenges, especially in low-resource settings (9, 28).

Health system-level inefficiencies, including fragmented service delivery and limited infrastructure, also restrict access (7, 29). Telemedicine has emerged as a compensatory mechanism to mitigate physical access constraints by enabling remote consultations and reducing dependence on geographic proximity. However, comparative evidence indicates that digital substitution does not eliminate structural inequities. In settings where digital infrastructure, accessibility standards, or healthcare integration are weak, telemedicine may shift rather than resolve existing access barriers. Thus, physical accessibility and digital accessibility should be considered complementary rather than interchangeable dimensions of inclusive healthcare (30–34).

#### 3.2.2 Attitudinal and sociocultural barriers

Attitudinal and sociocultural barriers were identified as pervasive determinants of healthcare inequity. Negative societal perceptions, stigma, and discrimination against individuals with disabilities were consistently reported across studies (4, 35–37). Healthcare provider attitudes also influenced care delivery, with implicit bias and assumptions contributing to unequal treatment and communication gaps (38, 39). Intersectional vulnerabilities, including gender and socioeconomic disparities, further compound access barriers (12, 35). Community-level stigma and cultural beliefs continue to influence care-seeking behaviors, particularly in LMIC and Middle Eastern contexts (9, 28). Studies emphasize that addressing attitudinal barriers requires not only policy interventions but also societal awareness and provider training (1, 23).

#### 3.2.3 Logistical and transportation barriers

Logistical barriers, particularly transportation challenges and geographic isolation, were widely reported across studies. Rural populations face significant challenges in reaching healthcare facilities due to distance, cost, and lack of reliable transport systems (6, 27, 40). Healthcare access is further hindered by fragmented referral systems and delays in service coordination, particularly in rural–urban healthcare linkages (40, 41). These challenges are exacerbated in LMIC settings where infrastructure limitations are more pronounced (24, 25). Telemedicine has been identified as a key intervention to reduce logistical constraints by eliminating the need for travel and enabling remote access to healthcare services (33, 42–44).

#### 3.2.4 Financial barriers

Financial barriers were consistently highlighted as a major constraint to healthcare access. High out-of-pocket expenditures, lack of insurance coverage, and indirect costs such as transportation and caregiving expenses limit service utilization (45–47). Disability-related costs, including assistive devices, rehabilitation, and long-term care, impose additional economic burden on individuals and families (48, 49). Studies indicate that financial hardship often leads to delayed or foregone care, particularly in LMICs (26, 50). Policy-level interventions such as health insurance expansion and social protection schemes have been shown to improve access and reduce inequities (45, 47, 51).

#### 3.2.5 Informational and communication barriers

Communication and informational barriers were identified as critical factors influencing healthcare quality and access. Language barriers, limited health literacy, and lack of accessible communication formats hinder effective patient-provider interaction (40, 52). Communication gaps are particularly evident among individuals with sensory or cognitive impairments, where a lack of interpreters or assistive communication tools restricts effective engagement (38, 39). Studies also highlight the importance of culturally competent communication in improving care outcomes (35, 37). Digital health tools, including telehealth platforms and translation technologies, have shown promise in addressing communication barriers and improving patient satisfaction (32, 52, 53). However, disparities in digital literacy and access remain limiting factors (54, 55).

#### 3.2.6 Policy and systemic barriers

Systemic barriers were identified across multiple studies as underlying determinants of inequitable healthcare access. These include weak governance structures, inadequate funding, fragmented service delivery, and inconsistent policy implementation (9, 28, 29). Despite global frameworks such as the CRPD, implementation at national and regional levels remains inconsistent, particularly in LMICs (21, 23, 24). Health system constraints such as workforce shortages and limited health information systems further exacerbate inequities (9, 26). Recent reforms, including Vision 2030 in Saudi Arabia, demonstrate efforts to strengthen healthcare systems through digitalization, workforce development, and infrastructure improvements (29, 56). However, these reforms should be interpreted cautiously because policy-level transformation does not automatically translate into equitable access for people with disabilities. Independent regional analyses continue to highlight implementation challenges, including workforce shortages, fragmented services, uneven access, and limited health information systems, suggesting that disability-inclusive outcomes depend on sustained monitoring, accessibility standards, and accountability mechanisms (9, 28). Similarly, broader MENA region analyses highlight ongoing challenges alongside gradual improvements in system performance (9). Across studies, a recurring tension was observed between policy commitment and implementation capacity. While international disability frameworks and digital health strategies increasingly promote inclusive healthcare models, practical implementation remains uneven because of differences in governance structures, workforce capacity, financing mechanisms, and technological infrastructure. This suggests that policy adoption alone is insufficient unless accompanied by operational resources and accountability mechanisms.

#### 3.2.7 Role of telemedicine and community-based strategies

Telemedicine and digital health interventions have emerged as important facilitators of healthcare access across multiple studies. These include teleconsultations, electronic health records, mobile health (mHealth), AI-driven systems, and virtual care platforms (30, 31, 56). Evidence from high-income countries (HICs) generally demonstrates moderate-to-strong improvements in access to specialist care and continuity of care, particularly for individuals with mobility limitations, whereas evidence from low- and middle-income countries (LMICs) remains mixed due to infrastructure constraints and inconsistent implementation capacity (33, 34, 43, 44, 57). However, comparative analysis across studies shows important inconsistencies in effectiveness depending on healthcare system readiness. While several HIC-based studies report reduced hospital visits and improved patient satisfaction, LMIC-based evidence highlights persistent access gaps due to unreliable connectivity, limited device availability, and weak digital literacy support systems, suggesting that the same intervention may produce unequal outcomes across contexts.

Notably, digital exclusion may persist even when basic access to devices and internet is available. For people with disabilities, barriers often arise from poor platform design rather than lack of connectivity. The distinction between accessibility and usability is therefore critical. Accessibility-focused studies (e.g., screen-reader compatibility interventions) report positive but limited improvements, whereas usability-focused evaluations show that even accessible platforms may fail in real-world use due to cognitive load, language complexity, or inadequate user training support (39, 58). This indicates that evidence strength is stronger for identifying barriers than for demonstrating long-term effectiveness of inclusive digital design interventions.

In addition, the included evidence suggests that telemedicine is more consistently reported as useful for follow-up care, triage, chronic disease monitoring, patient counseling, specialist access, and continuity of care, particularly where travel distance and mobility limitations restrict service use. In contrast, its role appears more variable in clinical contexts requiring physical examination, rehabilitation procedures, assistive-device fitting, complex diagnostics, or intensive face-to-face therapeutic interaction. This distinction was supported by studies describing benefits of telemedicine in remote access, chronic care, specialist consultation, and continuity of care, while also noting limitations related to diagnostic decision-making, usability, workflow integration, and services that require in-person assessment (34, 42, 43, 53, 57). Therefore, telemedicine should be interpreted as a context-dependent adjunct to healthcare delivery rather than a universal replacement for in-person care.

Community-based strategies, including community health workers, outreach programs, and home-based care, show more consistent effectiveness in LMIC settings compared to telemedicine, particularly in improving engagement, trust, and continuity of care. However, studies also report contradictory findings regarding scalability, with some programs demonstrating strong local success but limited sustainability when external funding or NGO support is withdrawn (16, 18, 59). Overall, evidence strength is moderate but context-sensitive, with stronger support for integration rather than stand-alone implementation. Together, integrated digital and community-based models appear to offer the greatest potential for improving healthcare access; however, the evidence does not support a universal model of implementation. Instead, effectiveness depends on alignment between intervention design and local conditions, including healthcare capacity, digital infrastructure, disability accessibility standards, socioeconomic characteristics, and community resources. The reviewed evidence therefore indicates that the central challenge is not simply expanding telemedicine or community programmes, but ensuring that these approaches are implemented within systems capable of supporting equitable and sustainable access. A key interpretive finding across the literature was that technology alone does not determine healthcare equity. Studies from high-resource settings often demonstrate benefits from mature digital ecosystems, whereas evidence from LMICs highlights that technological interventions frequently depend on broader social and infrastructural conditions. This difference illustrates that the effectiveness of telemedicine is relational rather than inherent: the same intervention may improve access in one setting while reinforcing exclusion in another.

## 4 Discussion

The findings of this narrative review highlight that barriers to healthcare access for people with disabilities are complex, interacting across structural, social, economic, and systemic levels. Consistent with prior global studies, disability-related inequities are not driven by a single determinant but rather by the cumulative effect of intersecting barriers that persist across both high-income and LMIC settings (1, 5, 11). These barriers are not only structural but also affect people’s experiences. Repeated exposure to inaccessible and non-inclusive healthcare settings can lead to stress, reduced independence, and reluctance to seek care. This often results in delayed diagnosis and poorer health outcomes. The findings of this review highlight how these barriers interact alongside emerging approaches such as telemedicine and community-based strategies. Compared with previous evidence syntheses, the present review supports and extends earlier findings. Hashemi et al. reported that people with disabilities in LMICs experience interacting attitudinal, informational, logistical, and systemic barriers to primary healthcare access, while Clemente et al. similarly identified communication, financial, organizational, and provider-related barriers across global healthcare settings. The findings of the present review are consistent with these conclusions, but extend them by linking disability-related healthcare barriers with telemedicine, community-based strategies, and regional health-system reforms, particularly in underserved and Arab contexts. Unlike earlier reviews that primarily focused on barriers to access, this review emphasizes that digital and community-based interventions can improve access only when supported by inclusive design, digital equity, workforce capacity, sustainable financing, and implementation accountability.

A common finding across the literature is the continued presence of physical and structural barriers within healthcare systems. Despite policy efforts and infrastructure improvements, many healthcare settings remain inaccessible or poorly adapted, which limits service use (21–23, 25). Evidence from rural and underserved settings shows that long distances and poor infrastructure make access to healthcare more difficult (6, 26, 27). In many cases, these barriers force people with disabilities to depend on caregivers for transport and navigating health services, which limits their independence and sense of dignity. Even in settings with disability laws in place, there are clear gaps between policy and actual practice (60). This suggests that health system reforms have not been fully implemented in ways that support inclusive service delivery, especially in LMICs and the Arab region (9, 28). These findings highlight the need for universal design, where healthcare environments are made accessible for all from the start, rather than adding adjustments later.

Beyond physical barriers, social and attitudinal factors also play a major role in limiting access to healthcare. Studies have reported stigma, discrimination, and negative attitudes from healthcare providers as key barriers to fair treatment (4, 36, 37). These issues affect both how people seek care and how providers respond to them. Research also shows that disability is often linked with other factors such as gender, poverty, and social disadvantage, which further increase exclusion (12, 35). For example, women with disabilities face additional challenges in accessing reproductive and maternal healthcare due to both physical inaccessibility and social norms. Evidence from LMICs shows that cultural beliefs and social attitudes continue to influence access to care, even where health systems have improved (24, 61). This suggests that policy changes alone are not enough, and efforts are also needed to change social attitudes and practices (1, 23).

Practical barriers, especially transport and distance, are another key issue, particularly in rural and low-resource settings. Studies show that long travel distances, high transport costs, and weak referral systems often delay or prevent access to care (6, 40). These challenges are reported in both LMICs and high-income countries, showing that location remains an important factor in access to healthcare (25, 27). Poor coordination between different levels of care also leads to delays and inefficiencies (40).

Financial barriers are also widely reported and are closely linked with poverty. High out-of-pocket costs, limited insurance coverage, and indirect costs such as caregiving reduce the ability of people with disabilities to access healthcare (45–47). Financial hardship is both a result of disability and a factor that worsens access, creating a cycle of disadvantage (26, 50). While policies such as health insurance and cash support programs can help, their impact is often limited by gaps in coverage and system weaknesses (45, 51). Caregivers also face financial and social pressure, which can further affect access to care (48, 49).

Informational and communication barriers further complicate access, especially for people with sensory or cognitive impairments. Differences in language, low health literacy, and lack of accessible formats make it harder for patients to understand and use healthcare services (40, 52). At the same time, many healthcare providers are not trained to communicate effectively with people with disabilities, which affects the quality of care (1, 39). The literature also highlights how communication challenges intersect with broader systemic and cultural factors, influencing trust, patient engagement, and health outcomes (35, 37).

At the policy and system level, the review identifies persistent gaps in governance, implementation, and resource allocation. Although international frameworks such as the CRPD provide a basis for disability-inclusive healthcare, their implementation remains inconsistent (21, 23). This gap between policy commitments and actual practice reflects weaknesses in enforcement, funding, and accountability. Health systems in LMICs and the MENA region face additional challenges, including workforce shortages, weak health information systems, and fragmented service delivery (9, 28). Recent initiatives, such as Saudi Arabia’s Vision 2030, indicate policy-level commitment to digital expansion, infrastructure development, and health-system reform (29, 56). However, reform agendas do not automatically ensure equitable implementation for people with disabilities. Independent regional evidence continues to identify persistent health-system challenges in the MENA region, including workforce shortages, fragmented service delivery, weak health information systems, and inequitable access, which may limit the practical impact of reforms unless disability-specific accessibility, monitoring, and accountability mechanisms are embedded into implementation (9, 28). Therefore, Vision 2030 and similar reform initiatives should be interpreted as important opportunities rather than definitive evidence of achieved disability-inclusive healthcare access.

This review also examines telemedicine and digital health as potential approaches for improving healthcare access among people with disabilities. Evidence indicates that telemedicine can reduce geographical barriers, decrease travel requirements, and improve access to specialist services, particularly for individuals living in remote areas or those experiencing mobility limitations (30, 31, 34). Its role expanded significantly during the COVID-19 pandemic, when remote consultations helped maintain continuity of care and reduced infection-related risks (33, 44). However, the findings suggest that the benefits of telemedicine are not universal and depend strongly on healthcare context, user characteristics, and system capacity.

Rather than eliminating healthcare inequalities, telemedicine may shift existing barriers into digital spaces. The digital divide should therefore be understood as a structural determinant of digital health equity rather than merely a technological limitation. Digital exclusion among people with disabilities results from the interaction of multiple factors, including functional limitations, socioeconomic disadvantage, educational inequalities, and geographical isolation. For example, a person with a disability living in a rural low-resource setting may experience simultaneous barriers related to inaccessible platforms, limited internet infrastructure, inability to afford digital devices, and reduced opportunities for digital skills development. This intersection of disability, poverty, and geography demonstrates that digital health inequalities reflect broader patterns of social exclusion. Individuals with disabilities may experience difficulties accessing platforms that lack accessibility features, while those living in low-resource settings may face challenges related to internet availability, device affordability, and digital skills (38, 39, 58). These challenges demonstrate that digital inclusion is a prerequisite for equitable telemedicine implementation. Importantly, improving digital inclusion requires attention to both accessibility and usability. Accessible design ensures that digital platforms can technically be used by people with different disabilities, whereas usability ensures that these technologies are practical, understandable, and acceptable within users’ daily lives. Failure to address either dimension may result in technologies that are available but ineffective for the populations they aim to serve. In addition, telemedicine may be less appropriate for healthcare needs requiring physical assessment, rehabilitation, assistive device fitting, or complex clinical decision-making (41, 42, 53–55). Therefore, remote healthcare should complement rather than replace accessible face-to-face services.

The effectiveness of telemedicine is also influenced by health system factors, including provider preparedness, reimbursement structures, data governance, and regulatory frameworks. Rapid adoption during the pandemic highlighted implementation challenges related to workflow integration, patient-provider communication, and long-term sustainability (38, 53). Therefore, telemedicine should be viewed as one component of disability-inclusive healthcare rather than a standalone solution. Sustainable implementation requires investment in accessible digital infrastructure, provider training, supportive policies, and integration with community-based services.

Community-based approaches are also important in improving access to healthcare. Evidence shows that community health workers and outreach programs can reduce gaps in access, especially in underserved areas (16, 18, 59). These approaches help connect formal healthcare systems with local communities and address issues related to access, awareness, and trust. However, their success depends on proper training, integration into health systems, and continued funding (18).

The findings suggest that no single solution can address the complex barriers faced by people with disabilities. A combined approach is needed, including health system improvements, digital tools, community-based support, and social policies. These elements must work together. For example, financial support is only effective if services are accessible, and digital solutions must be supported by inclusive policies.

This review also highlights the importance of context. Barriers and solutions differ across settings due to differences in economic conditions, cultural norms, and health system capacity (11, 61). Therefore, interventions must be tailored to local contexts rather than relying on standard models.

### 4.1 Obstacles and considerations

Although this narrative review offers vital insights into the obstacles that prevent persons with disabilities from gaining access to healthcare and emphasizes the role that telehealth, community health, and socioeconomic assistance play, it is important to realize that there are various limitations that must be taken into consideration in order to present a balanced viewpoint.

#### 4.1.1 The absence of quantitative content analysis

On the other hand, this narrative review does not include any quantitative analysis or meta-synthesis, which is a significant shortcoming. Narrative reviews, in contrast to systematic reviews, do not collect data in order to evaluate the extent of problems or the effectiveness of treatments. The conclusion that can be drawn from this analysis is that it is not possible to offer accurate estimations of the prevalence of obstacles or the demonstrable effect of community-based solutions and telemedicine.

#### 4.1.2 Subjective considerations in the selection of sources

Subjectivity is inevitably involved in the process of selecting research and sources through which narrative evaluations are conducted. There is a possibility of bias in the selection of literature, which might possibly have an effect on the findings that are derived, despite the fact that attempts were made to cover a wide variety of research. Given the extensive range of challenges and potential solutions that have been mentioned, this constraint is of utmost significance. It is possible that some topics may be overrepresented or underexplored owing to variances in the research that is now accessible.

#### 4.1.3 Variability in telehealth and community health solutions

The effectiveness of telemedicine and community-based interventions varies considerably depending on local healthcare infrastructure, socioeconomic conditions, digital accessibility, workforce capacity, and regulatory environments (39, 58). While telemedicine can reduce geographic and logistical barriers, its benefits may be limited in settings with inadequate internet connectivity, limited digital literacy, or inaccessible technology. Similarly, community-based programmes require sustained funding, workforce support, and integration within formal healthcare systems to achieve long-term impact (18). Therefore, these interventions should be adapted to local needs rather than implemented through uniform models across different healthcare contexts.

## 5 Conclusion

This narrative review highlights the complex and persistent barriers that people with disabilities face in accessing healthcare across diverse settings. These barriers are more pronounced in resource-limited contexts due to infrastructural and sociocultural challenges. Telemedicine and community-based approaches show promise in improving access, but their success depends on inclusive design and effective implementation. Addressing these challenges requires coordinated efforts across policy, practice, and research to ensure healthcare systems are accessible, equitable, and responsive to the needs of people with disabilities.

## 6 Strengths and limitations

This narrative review provides a broad synthesis of global evidence, highlighting structural, social, and health-system barriers alongside emerging solutions such as telemedicine and community-based approaches. Its strength lies in integrating evidence from diverse healthcare contexts to provide a comprehensive understanding of disability-inclusive healthcare access. However, several methodological limitations should be acknowledged. As a narrative review, this study does not follow a systematic review protocol and therefore does not include protocol registration, exhaustive reproducibility of search decisions, or quantitative synthesis of findings. Unlike systematic reviews, narrative reviews are inherently interpretive and do not allow estimation of effect sizes or comparative statistical evaluation of intervention effectiveness. A further limitation is the absence of formal risk-of-bias assessment or standardized quality appraisal tools (e.g., CASP, ROBINS-I). As a result, the included studies were not weighted according to methodological rigor, which may influence the strength and reliability of synthesized conclusions. The review may be affected by publication bias, as studies reporting positive outcomes of telemedicine or community interventions are more likely to be published compared to studies reporting null or negative findings. This may lead to an overrepresentation of successful intervention outcomes in the synthesized evidence base.

This limitation is particularly relevant to telemedicine because successful digital health initiatives may be more visible in the published literature than unsuccessful, incomplete, or poorly implemented programs. As a result, the apparent feasibility, acceptability, and effectiveness of telemedicine may be overestimated, especially for people with disabilities living in rural or low-resource settings where connectivity, device access, digital literacy, and accessibility features remain uneven. Therefore, conclusions regarding telemedicine should be interpreted cautiously and viewed as context-dependent rather than universally generalizable.

The inclusion of English-language studies only introduces potential language bias. Relevant studies published in other languages, particularly from non-English-speaking LMICs, may have been excluded, limiting the comprehensiveness of global evidence representation. Focusing primarily on literature from 2015 to 2025 introduces a timeframe bias, with increased representation of COVID-19-era telemedicine studies. These findings may reflect short-term emergency adaptations rather than long-term sustainable healthcare models, which limit generalisability to post-pandemic healthcare systems. The included literature shows uneven geographical distribution, with stronger representation from high-income countries and selected LMIC regions (e.g., the Middle East and Sub-Saharan Africa). Additionally, there is an overrepresentation of observational and review-based studies compared to interventional or longitudinal research, which limits causal inference. The inclusion of diverse study designs (qualitative, quantitative, policy, and conceptual papers) introduces methodological heterogeneity, making direct comparison difficult and limiting the ability to draw standardized conclusions across interventions.

The limited availability of high-quality evidence from some low- and middle-income countries (LMICs), particularly regarding long-term outcomes of telemedicine and community-based interventions, restricts generalizability. Furthermore, prioritizing literature from the last decade enabled the review to capture recent developments in digital health; however, it may have increased representation of COVID-19-era telemedicine studies, which may reflect short-term emergency adaptations rather than sustainable long-term models. Earlier foundational studies were included where relevant, although some historical evidence may not have been captured. Further context-specific and methodologically rigorous research is required.

## References

[1] Clemente KAP Silva SVD Vieira GI Bortoli MCD Toma TS Ramos VD . Barriers to the access of people with disabilities to health services: a scoping review. Rev Saude Publica. (2022) 56:64. doi: 10.11606/s1518-8787.2022056003893, 35792776 PMC9239543

[2] World Health Organization. *Global Report on Health Equity for Persons with Disabilities*. (2022). Available online at: https://www.who.int/teams/noncommunicable-diseases/sensory-functions-disability-and-rehabilitation/global-report-on-health-equity-for-persons-with-disabilities (Accessed February 1, 2026).

[3] Centers for Disease Control and Prevention. *Disability and Health: Information for Health Care Providers*. Centers for Disease Control and Prevention (2020). Available online at: https://www.cdc.gov/ncbddd/disabilityandhealth/hcp.html.

[4] Barbareschi G Carew MT Johnson EA Kopi N Holloway C. “When they see a wheelchair, they’ve not even seen me”—factors shaping the experience of disability stigma and discrimination in Kenya. Int J Environ Res Public Health. (2021) 18:4272. doi: 10.3390/ijerph18084272, 33920601 PMC8073617

[5] Mcbride-Henry K Nazari Orakani S Good G Roguski M Officer TN. Disabled people’s experiences accessing healthcare services during the Covid-19 pandemic: a scoping review. BMC Health Serv Res. (2023) 23:346. doi: 10.1186/s12913-023-09336-4, 37024832 PMC10078067

[6] Dassah E Aldersey H Mccoll MA Davison C. Factors affecting access to primary health care services for persons with disabilities in rural areas: a “best-fit” framework synthesis. Glob Health Res Policy. (2018) 3:36. doi: 10.1186/s41256-018-0091-x PMC6305566 30603678

[7] Panezai S Ahmad MM Saqib SE. Factors affecting access to primary health care services in Pakistan: a gender-based analysis. Dev Pract. (2017) 27:813–27. doi: 10.1080/09614524.2017.1344188

[8] Eissa Saad MA Borowska-Beszta B. Disability in the Arab world: a comparative analysis within culture. Psycho Educ Res Rev. (2019) 8:29–47.

[9] Katoue MG Cerda AA García LY Jakovljevic M. Healthcare system development in the Middle East and North Africa region: challenges, endeavors and prospective opportunities. Front Public Health. (2022) 10:1045739. doi: 10.3389/fpubh.2022.1045739, 36620278 PMC9815436

[10] Jiang S Musa M. Evolving narratives of gulf press representations of women with disabilities. J Int Womens Stud. (2024) 26:10.

[11] Hashemi G Wickenden M Bright T Kuper H. Barriers to accessing primary healthcare services for people with disabilities in low and middle-income countries, a Meta-synthesis of qualitative studies. Disabil Rehabil. (2022) 44:1207–20. doi: 10.1080/09638288.2020.1817984, 32956610

[12] Rotarou ES Sakellariou D. Inequalities in access to health care for people with disabilities in Chile: the limits of universal health coverage. Crit Public Health. (2017) 27:604–16. doi: 10.1080/09581596.2016.1275524

[13] Mulumba M Nantaba J Brolan CE Ruano AL Brooker K Hammonds R. Perceptions and experiences of access to public healthcare by people with disabilities and older people in Uganda. Int J Equity Health. (2014) 13:76. doi: 10.1186/s12939-014-0076-4, 25928896 PMC4188877

[14] Ezeamii VC Okobi OE Wambai-Sani H Perera GS Zaynieva S Okonkwo CC . Revolutionizing healthcare: how telemedicine is improving patient outcomes and expanding access to care. Cureus. (2024) 16:e63881. doi: 10.7759/cureus.63881, 39099901 PMC11298029

[15] Muzzamil M Karim M Khurram SS Aga IZ Hashmi S Hassan HG . Patient perspectives and utilization of telemedicine for chronic conditions in Pakistan barriers and facilitators towards its access. Discov Public Health. (2025) 22:30. doi: 10.1186/s12982-025-00409-9

[16] Khatri RB Endalamaw A Erku D Wolka E Nigatu F Zewdie A . Enablers and barriers of community health programs for improved equity and universal coverage of primary health care services: a scoping review. Bmc Prim Care. (2024) 25:385. doi: 10.1186/s12875-024-02629-5, 39472794 PMC11520389

[17] Kwemoi K. The role of community health workers in bridging gaps in access to care. Eur Exp J Sci Appl Res. (2024) 6:1–7.

[18] Shrestha P Afsana K Weerasinghe MC Perry HB Joshi H Rana N . Strengthening primary health care through community health workers in South Asia. Lancet Reg Health Southeast Asia. (2024) 28:100463. doi: 10.1016/j.lansea.2024.100463, 39301268 PMC11410731

[19] Wong G Greenhalgh T Westhorp G Buckingham J Pawson R. Rameses publication standards: Meta-narrative reviews. J Adv Nurs. (2013) 69:987–1004. doi: 10.1111/jan.12092, 23356699

[20] Popay J Roberts H Sowden A Petticrew M Arai L Rodgers M . Guidance on the Conduct of Narrative Synthesis in Systematic Reviews: A Product from the Esrc Methods Programme Version. Lancaster: Lancaster University (2006). p. 92.

[21] Eide AH Mannan H Khogali M Van Rooy G Swartz L Munthali A . Perceived barriers for accessing health services among individuals with disability in four African countries. PLoS One. (2015) 10:e0125915. doi: 10.1371/journal.pone.0125915, 25993307 PMC4489521

[22] Grills N Singh L Pant H Varghese J Murthy G Hoq M . Access to services and barriers faced by people with disabilities: a quantitative survey. Disabil CBR Incl Dev. (2017) 28:23–3. doi: 10.5463/dcid.v28i2.615

[23] Marella M Smith F Hilfi L Sunjaya DK. Factors influencing disability inclusion in general eye health services in Bandung, Indonesia: a qualitative study. Int J Environ Res Public Health. (2018) 16:23. doi: 10.3390/ijerph16010023, 30583466 PMC6338889

[24] Munthali AC Swartz L Mannan H Maclachlan M Chilimampunga C Makupe C. “This one will delay us”: barriers to accessing health care services among persons with disabilities in Malawi. Disabil Rehabil. (2019) 41:683–90. doi: 10.1080/09638288.2017.1404148, 29172751

[25] Tesfaye T Woldesemayat EM Chea N Wachamo D. Accessing healthcare Services for People with physical disabilities in Hawassa City administration, Ethiopia: a cross-sectional study. Risk Manag Healthc Policy. (2021) 14:3993–4002. doi: 10.2147/RMHP.S317849, 34602827 PMC8478668

[26] Coombs NC Meriwether WE Caringi J Newcomer SR. Barriers to healthcare access among U.S. adults with mental health challenges: a population-based study. SSM Popul Health. (2021) 15:100847. doi: 10.1016/j.ssmph.2021.100847, 34179332 PMC8214217

[27] Oluyede L Cochran AL Wolfe M Prunkl L Mcdonald N. Addressing transportation barriers to health care during the Covid-19 pandemic: perspectives of care coordinators. Transp Res Part A Policy Pract. (2022) 159:157–68. doi: 10.1016/j.tra.2022.03.010, 35283561 PMC8898700

[28] Kronfol N. Access and barriers to health care delivery in Arab countries: a review. East Mediterr Health J. (2012) 18:1239–46. doi: 10.26719/2012.18.12.1239 23301399

[29] Mani ZA Goniewicz K. Transforming healthcare in Saudi Arabia: a comprehensive evaluation of vision 2030’s impact. Sustainability. (2024) 16:3277. doi: 10.3390/su16083277

[30] Anawade PA Sharma D Gahane S. A comprehensive review on exploring the impact of telemedicine on healthcare accessibility. Cureus. (2024) 16. doi: 10.7759/cureus.55996, 38618307 PMC11009553

[31] Haleem A Javaid M Singh RP Suman R. Telemedicine for healthcare: capabilities, features, barriers, and applications. Sens Int. (2021) 2:100117. doi: 10.1016/j.sintl.2021.100117, 34806053 PMC8590973

[32] Hwei L Octavius G. Potential advantages and disadvantages of telemedicine: a literature review from the perspectives of patients, medical personnel, and hospitals. J Commun Empowerment Health. (2021) 4:228–186. doi: 10.22146/jcoemph.64247

[33] Portnoy J Waller M Elliott T. Telemedicine in the era of Covid-19. J Allergy Clin Immunol Pract. (2020) 8:1489–91. doi: 10.1016/j.jaip.2020.03.008, 32220575 PMC7104202

[34] Shigekawa E Fix M Corbett G Roby DH Coffman J. The current state of telehealth evidence: a rapid review. Health Aff (Millwood). (2018) 37:1975–82. doi: 10.1377/hlthaff.2018.05132, 30633674

[35] Kabia E Mbau R Muraya KW Morgan R Molyneux S Barasa E. How do gender and disability influence the ability of the poor to benefit from pro-poor health financing policies in Kenya? An intersectional analysis. Int J Equity Health. (2018) 17:149. doi: 10.1186/s12939-018-0853-6 PMC6146517 30231887

[36] Sahu KK Sahu S. Attitudinal barrier experienced by people with disabilities. J Disabil Stud. (2015) 1:53–4.

[37] Soltani S Takian A Akbari Sari A Majdzadeh R Kamali M. Cultural barriers in access to healthcare services for people with disability in Iran: a qualitative study. Med J Islam Repub Iran. (2017) 31:293–9. doi: 10.14196/mjiri.31.51, 29445680 PMC5804431

[38] Khoong E Nouri S Lyles C Karliner L. *Addressing Equity in Telemedicine for Chronic Disease Management During the Covid-19 Pandemic*. (2020).

[39] Valdez RS Rogers CC Claypool H Trieshmann L Frye O Wellbeloved-Stone C . Ensuring full participation of people with disabilities in an era of telehealth. J Am Med Inform Assoc. (2021) 28:389–92. doi: 10.1093/jamia/ocaa297, 33325524 PMC7717308

[40] Wilson MM Devasahayam AJ Pollock NJ Dubrowski A Renouf T. Rural family physician perspectives on communication with urban specialists: a qualitative study. BMJ Open. (2021) 11:e043470. doi: 10.1136/bmjopen-2020-043470, 33986048 PMC8126282

[41] Veronica A. Using telemedicine in clinical decision-making. Pract Nurs. (2014) 25:42–6. doi: 10.12968/pnur.2014.25.1.42

[42] Rutledge CM Kott K Schweickert PA Poston R Fowler C Haney TS. Telehealth and eHealth in nurse practitioner training: current perspectives. Adv Med Educ Pract. (2017) 8:399–409. doi: 10.2147/AMEP.S116071, 28721113 PMC5498674

[43] Serper M Volk ML. Current and future applications of telemedicine to optimize the delivery of Care in Chronic Liver Disease. Clin Gastroenterol Hepatol. (2018) 16:157–161.e8. doi: 10.1016/j.cgh.2017.10.004, 29389489 PMC6334286

[44] Shokri F Bahrainian S Tajik F Rezvani E Shariati A Nourigheimasi S . The potential role of telemedicine in the infectious disease pandemic with an emphasis on Covid-19: a narrative review. Health Sci Rep. (2023) 6:e1024. doi: 10.1002/hsr2.1024, 36620507 PMC9811063

[45] Jamal MH Abdul Aziz AF Aizuddin AN Aljunid SM. Successes and obstacles in implementing social health insurance in developing and middle-income countries: a scoping review of 5-year recent literatures. Front Public Health. (2022) 10:918188. doi: 10.3389/fpubh.2022.918188, 36388320 PMC9648174

[46] Mitra S Palmer M Kim H Mont D Groce N. Extra costs of living with a disability: a review and agenda for research. Disabil Health J. (2017) 10:475–84. doi: 10.1016/j.dhjo.2017.04.007, 28501322

[47] Zhang X Ma L Liu X Cui N Guo B Zhang L. Association between health insurance programs and rehabilitation services utilisation among people with disabilities: evidence from China. Public Health. (2024) 232:201–7. doi: 10.1016/j.puhe.2024.04.025, 38815542

[48] Lamech N Raghuraman S Vaitheswaran S Rangaswamy T. The support needs of family caregivers of persons with dementia in India: implications for health services. Dementia. (2019) 18:2230–43. doi: 10.1177/1471301217744613, 29199451

[49] Thomas HMC Phan CT Phillips C Wan S Doyon K Gray T . Ethical and policy implications of financial burden in family caregivers. J Hosp Palliat Nurs. (2022) 24:e226–32. doi: 10.1097/NJH.0000000000000887, 35666768 PMC10365074

[50] Cruz RCS Moura LBA Soares Neto JJ. Conditional cash transfers and the creation of equal opportunities of health for children in low and middle-income countries: a literature review. Int J Equity Health. (2017) 16:161. doi: 10.1186/s12939-017-0647-2, 28859650 PMC5580215

[51] National Academies of Sciences, Engineering, and Medicine; Health and Medicine Division; Board on Population Health and Public Health Practice; Committee on Community-Based Solutions to Promote Health Equity in the United States. Communities in Action: Pathways to Health Equity. Baciu A Negussie Y Geller A Weinstein JN, editors. Washington (DC): National Academies Press (US) (2017). 28418632

[52] Al Shamsi H Almutairi AG Al Mashrafi S Al Kalbani T. Implications of language barriers for healthcare: a systematic review. Oman Med J. (2020) 35:e122. doi: 10.5001/omj.2020.40, 32411417 PMC7201401

[53] Sauers-Ford HS Hamline MY Gosdin MM Kair LR Weinberg GM Marcin JP . Acceptability, usability, and effectiveness: a qualitative study evaluating a pediatric telemedicine program. Acad Emerg Med. (2019) 26:1022–33. doi: 10.1111/acem.13763, 30974004 PMC6732030

[54] Edwards L Thomas C Gregory A Yardley L O'cathain A Montgomery AA . Are people with chronic diseases interested in using telehealth? A cross-sectional postal survey. J Med Internet Res. (2014) 16:e123. doi: 10.2196/jmir.3257, 24811914 PMC4034113

[55] Henderson K Davis T Smith M King M. Nurse practitioners in telehealth: bridging the gaps in healthcare delivery. J Nurse Pract. (2014) 10:845–50. doi: 10.1016/j.nurpra.2014.09.003

[56] Alwatban N Othman F Almosnid N Alkadi K Alajaji M Aldeghaither D. "The Emergence and Growth of Digital Health in Saudi Arabia: A Success Story". In: Kozlakidis Z Muradyan A Sargsyan K, editors. Digitalization of Medicine in Low- and Middle-Income Countries. Sustainable Development Goals Series. Cham: Springer (2024)

[57] Pappas Y Vseteckova J Mastellos N Greenfield G Randhawa G. Diagnosis and decision-making in telemedicine. J Patient Exp. (2019) 6:296–304. doi: 10.1177/2374373518803617, 31853485 PMC6908983

[58] Annaswamy TM Verduzco-Gutierrez M Frieden L. Telemedicine barriers and challenges for persons with disabilities: Covid-19 and beyond (2020) 13:100973. doi: 10.1016/j.dhjo.2020.100973, 32703737 PMC7346769

[59] World Health Organization. Community Health Workers: A Strategy to Ensure Access to Primary Health Care Services. Geneva: World Health Organization (2016).

[60] Ordway A Garbaccio C Richardson M Matrone K Johnson KL. Health care access and the Americans with disabilities act: a mixed methods study. Disabil Health J. (2021) 14:100967. doi: 10.1016/j.dhjo.2020.100967, 32768336

[61] Zuurmond M Mactaggart I Kannuri N Murthy G Oye JE Polack S. Barriers and facilitators to accessing health services: a qualitative study amongst people with disabilities in Cameroon and India. Int J Environ Res Public Health. (2019) 16:1126. doi: 10.3390/ijerph16071126, 30934813 PMC6480147

